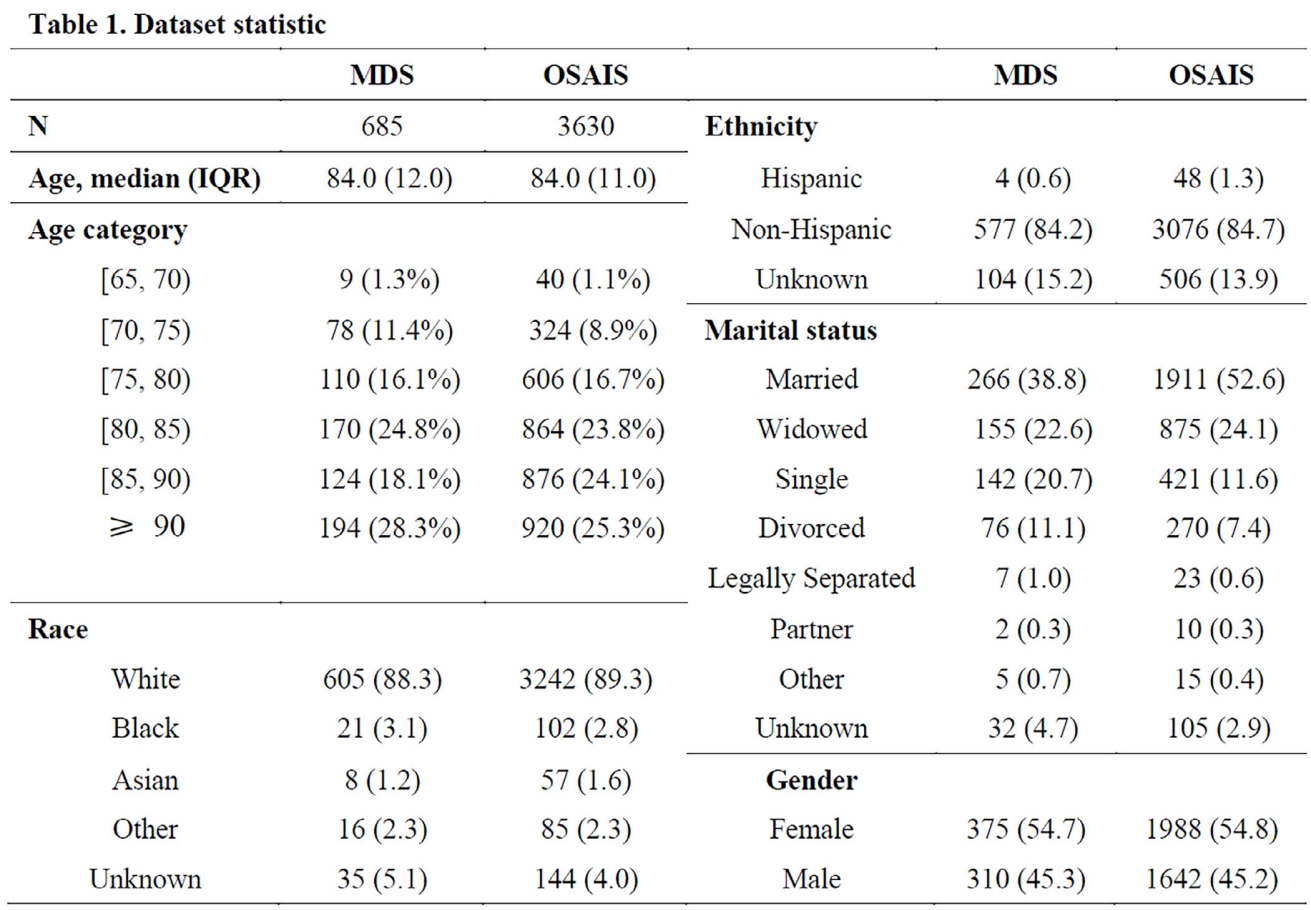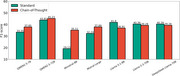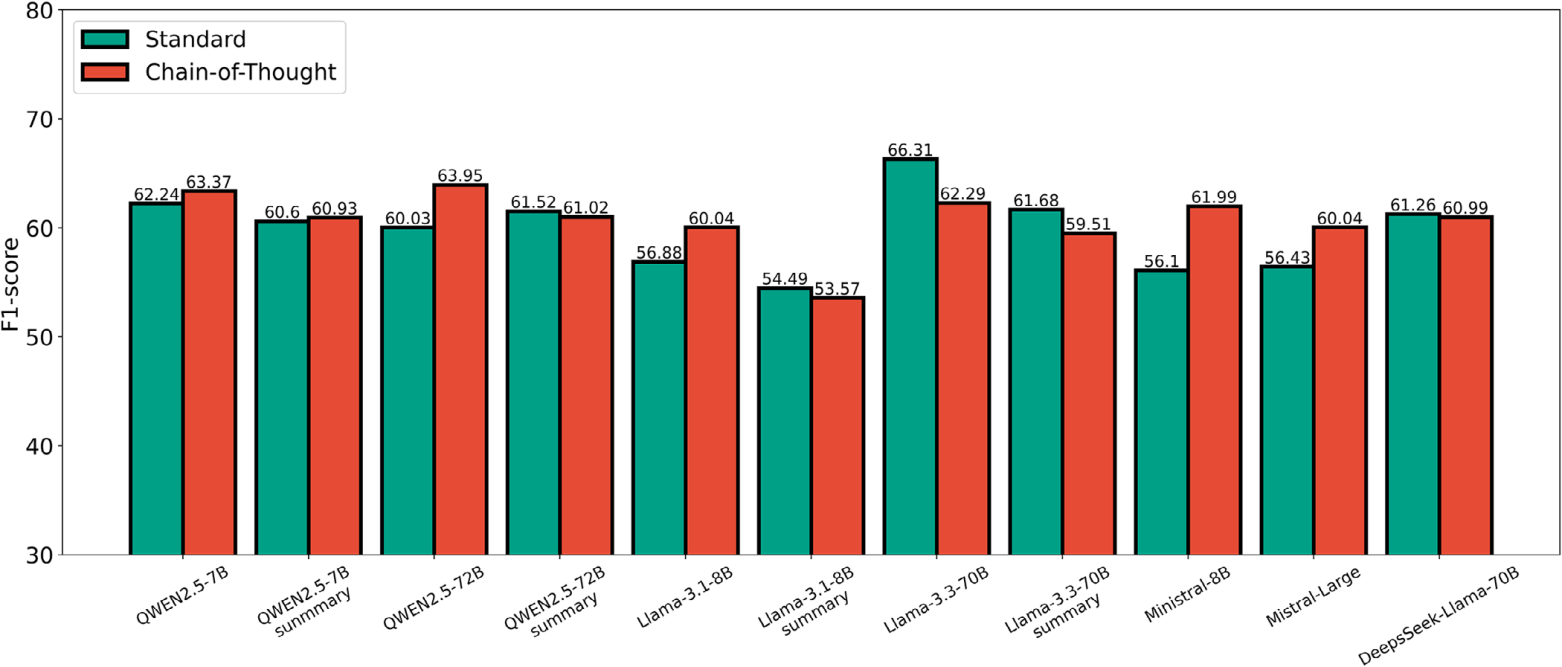# Cognitive Assessment of Dementia Patients Using Large Language Models

**DOI:** 10.1002/alz70858_103769

**Published:** 2025-12-25

**Authors:** Jiageng Wu, Richard Wyss, Josh Lin, Jie Yang

**Affiliations:** ^1^ Brigham and Women's Hospital, Boston, MA, USA

## Abstract

**Background:**

Cognitive function is a key determinant of daily functioning and quality of life in dementia patients. Accurate and timely cognitive assessments are critical for disease management, risk stratification, and effective intervention implementation. However, traditional assessments often require significant resources, limiting their scalability. Large language models (LLMs) offer a novel approach to predicting cognitive function by leveraging unstructured data from Electronic Health Records (EHRs).

**Method:**

This retrospective cohort study analyzed EHRs of patients aged ≥65 years diagnosed with dementia between 2013 and 2020 at Mass General Brigham (MGB). Cognitive function was evaluated using the Brief Interview for Mental Status (BIMS) from the Minimum Data Set (MDS) or M1700 Cognitive Function from the Outcome and Assessment Information Set (OASIS). The clinical note from the past year were used as model input. Multiple state‐of‐the‐art LLMs were tested, including QWEN2.5 (7B and 72B), Llama 3.1 (8B), Llama 3.3 (70B), Ministral‐8B, Mistral Large, and DeepSeek‐Llama (70B). Three inference strategies were explored: (1) Standard mode, directly outputting predictions; (2) Chain‐of‐Thought (CoT) mode, generating step‐by‐step reasoning; and (3) Summary‐based mode, summarizing clinical notes before integrated prediction. Model performance was evaluated using F1‐scores with bootstrapping.

**Result:**

The study included 685 patients who underwent MDS‐cognitive and 3,630 patients assessed using OASIS. Overall, LLMs demonstrated strong cross‐dataset generalizability. Among them, Llama‐3.3‐70B achieved the highest F1‐score (66.31%) in OASIS assessments, while QWEN2.5‐72B performed best in MDS (45.23%). The larger LLMs provided significant performance gains, whereas the CoT and summary strategies enhanced interpretability but did not consistently improve accuracy. This may be due to the models' limited understanding of clinical text, making precise information extraction and reasoning challenging.

**Conclusion:**

This study highlights the potential of LLMs in predicting cognitive function for dementia patients using EHRs. Larger LLMs showed superior performance, while the CoT and summary strategies improved interpretability but may slightly impair performance. The results highlight the necessity for further adoption and optimization of LLM modeling and reasoning strategies to better handle clinical text, minimizing hallucinations and improving reliability. Integrating LLMs into cognitive assessment workflows could enhance efficiency and accessibility, supporting more effective dementia care and management.